# Contemporary biomedical engineering perspective on volitional evolution for human radiotolerance enhancement beyond low-earth orbit

**DOI:** 10.1093/synbio/ysab023

**Published:** 2021-09-02

**Authors:** Alexander M Borg, John E Baker

**Affiliations:** Departments of Biomedical Engineering and Radiation Oncology, Wake Forest University, Winston-Salem, NC, USA; Radiation Biosciences Laboratory, Medical College of Wisconsin, Milwaukee, WI, USA

**Keywords:** volitional evolution, biomedical engineering modeling, ionizing space radiation, interplanetary travel, human engineering ethics

## Abstract

A primary objective of the National Aeronautics and Space Administration (NASA) is expansion of humankind’s presence outside low-Earth orbit, culminating in permanent interplanetary travel and habitation. Having no inherent means of physiological detection or protection against ionizing radiation, humans incur capricious risk when journeying beyond low-Earth orbit for long periods. NASA has made large investments to analyze pathologies from space radiation exposure, emphasizing the importance of characterizing radiation’s physiological effects. Because natural evolution would require many generations to confer resistance against space radiation, immediately pragmatic approaches should be considered. Volitional evolution, defined as humans steering their own heredity, may inevitably retrofit the genome to mitigate resultant pathologies from space radiation exposure. Recently, uniquely radioprotective genes have been identified, conferring local or systemic radiotolerance when overexpressed *in vitro* and *in vivo*. Aiding in this process, the CRISPR/Cas9 technique is an inexpensive and reproducible instrument capable of making limited additions and deletions to the genome. Although cohorts can be identified and engineered to protect against radiation, alternative and supplemental strategies should be seriously considered. Advanced propulsion and mild synthetic torpor are perhaps the most likely to be integrated. Interfacing artificial intelligence with genetic engineering using predefined boundary conditions may enable the computational modeling of otherwise overly complex biological networks. The ethical context and boundaries of introducing genetically pioneered humans are considered.

## 1. Introduction

### 1.1 NASA directorates and ionizing space radiation

A primary objective of the National Aeronautics and Space Administration (NASA) is the human exploration and operations mission directorate, providing NASA the authority and capability to conduct research pertaining to human exploration beyond low-Earth orbit (1). NASA’s Perseverance Rover is presently testing technologies to help prepare for an extended human presence on Mars, and NASA has fueled extraordinary analyses in quantifying and mitigating the physiological effects of ionizing radiation exposure in space (2, 3). Outside Earth’s protective magnetosphere, deep space harbors both omnipresent galactic cosmic radiation (GCR) and spontaneous events like Solar Particle Events (SPEs) and coronal mass ejections (CMEs) (4, 5). Despite developments in the prediction of and protection against spontaneous events, sustained cosmonaut exposure to GCR remains unavoidable. The most common sources of space radiation are described in Table 1.

**Table 1.** Properties and variables of common sources of radiation, toxic to humans beyond Earth’s magnetosphere

Astronomical consideration	Foreseeability	Duration	Constituency	Primary factors
GCR	Known (13)	Perpetual (14)	87% protons, 12% α-particles, 1% HZE ions (10)	Solar cycle (15)
Solar flare (19)	Unforeseeable but improving (16)	Minutes to hours (17)	Mostly photons (18)	Solar cycle, vicinity to sun (13)
CME	Modest 3-day forecast available (20)	Several hours (5)	Protons, electrons and HZE ions (21)	Size, speed and direction of CME (22)
SPE	Likely similar to that for CMEs (23)	Seconds to hours (17)	Mostly protons, some electrons and HZE ions (24)	Solar cycle, otherwise obscure (4)

Two physical mechanisms of particle acceleration in deep space result in two types of random events: impulsive and gradual. Impulsive events (e.g. solar flares) are typically rich in Helium-3 and electrons and are associated with radio bursts and x-ray flares. Gradual events (e.g. shockwaves from CMEs) involve largely protons and occur with less frequency (6). Discrepancies in dose rates and particle types result in drastically diverse acute and chronic pathologies, limiting the extensibility of existing studies (7–9).

### 1.2 Space radiation complications

According to NASA, every cell in an astronaut’s body is traversed by a proton, a helium nucleus and a high atomic number and energy nucleus about once every few days, weeks and months, respectively, due to GCR alone. This corresponds to tissue doses and effective dose-rates of about 0.3–0.6 mGy/day and 1–1.8 mSv/day, respectively, and, although difficult to scale, this translates to ∼0.09–0.18 single-strand DNA breaks and 0.009–0.018 double-strand DNA breaks per cell per day (10, 11). These breaks occur both directly from irradiation and indirectly from free radicals produced by intracellular water molecules (12). On the shortest possible return mission to Mars, staying 30 days on the surface, a cosmonaut would absorb more than 500 mGy equivalent over the 650 day mission, empirically suggested as the dose threshold to induce cataracts requiring surgery in Japanese atomic bomb survivors (8, 25). Age at exposure to radiation is a primary determinant of outcomes, imposing higher risk at younger ages (26–31). Although clinically overshadowed by solid cancers and difficult to quantify, the long-term quality and expectancy of an astronaut’s life may be irreversibly impacted by functional degradation of, among others, the musculoskeletal, nervous and cardiovascular systems, in a remarkably short timeframe (10, 32, 33). A positive feedback cycle in which chronic stress from radiation further reduces an individual’s psychological ability to cope with cancer may even manifest (34).

Preventing long-term radiation-induced damage is crucial to protect humans during interplanetary travel and while living on Mars, even with fabricated shielding from the local Martian regolith. Because ubiquitous physical shielding transported from earth to Mars is not feasible due to its high cost and weight (accentuated by gravity during takeoff), additional measures must be considered (35). An alternative has emerged only recently as potentially viable and arguably fundamental: steering human evolution as a means of providing a genetic shield against radiation damage. Our central aim is to demonstrate that such human engineering deserves viable consideration alongside other potential solutions to protect astronauts from the effects of space radiation exposure.

## 2. Background: genetic differentiation

### 2.1 Natural human adaptation

The human genome is continuously evolving, and contemporary global studies show strong evidence of convergent human evolution with respect to our population’s nutritional, geographical and pathological environments (36–40). For example, the Nunavik Inuit in Quebec, Canada, have genetically adapted to a diet of about 75% ingested animal fat (41). Other populations have evolved independently to live in regions over 4 km above sea level (e.g. the Tibetan Plateau, Andean Altiplano and Ethiopian Highlands), genetically adapted to threats of hypoxia, extreme day-to-night temperature fluctuations and chronic conditions from abnormal oxygen saturation of hemoglobin (42–47). Because *Homo sapiens* evolved in an environment perpetually isolated from GCR, no defense structures are extant in the human body to protect against its sudden introduction. Furthermore, mammalian physiological systems neither harbor receptors triggered specifically by ionizing radiation nor have precise or ubiquitous detection mechanisms attributed to its exposure; the innate immune response serves as the primary conduit for detecting resultant tissue damage from the exposure (32, 48, 49). Because an astronaut’s constant exposure to space radiation is teratogenic, conventional human reproduction and fetal development would not be evolutionarily sustainable (50).

### 2.2 CRISPR/Cas9 genetic engineering and limitations

In 2015, the expensive, imprecise and relatively inconsistent methods of genetically altering animal zygotes were superseded by the clustered regularly interspaced short palindromic repeat (CRISPR)-associated system (Cas) to manipulate DNA (51, 52). The so-called CRISPR/Cas9 technique revolutionized genomic alternation, and scientists have already proven it effective in both knocking-out (by non-homologous end joining) and knocking-in (by homology directed repair) genes in the zygotes of *Homo sapiens* as well as other organisms, such as zebrafish, rats and mice (53–60). Still, unexpected, partial genetic similarities sometimes result in CRISPR-mediated cleavage at off-target locations; the frequency and obviousness of such mismatches are functions of myriad factors, such as local and global DNA positioning, sequence homology and Cas9 expression level. The potential of these off-target activities are crucial shortcomings in the CRISPR system, manifesting as undesired mosaicism and mutation (61, 62). Additionally, employing the CRISPR/Cas9 technique for safe human transgenesis would likely require thousands of secondary and tertiary nucleotide modifications per genome per cell without germline engineering (63). Beyond these challenges, others pose risk for truly effective and safe transportation of CRISPR/Cas9 plasmids, such as mutagenesis, carcinogenesis and immunogenicity complications resulting from the nature of the viral vector (64–68). Despite such inherent limitations, contemporary innovations indicate that CRISPR and next-generation technologies have the potential to accomplish volitional evolution in a foreseeable timeframe (69).

### 2.3 Ethics of human engineering

Many progenitor-cell-based strategies are evolving rapidly alongside CRISPR to achieve this goal, such as *in vitro* gametogenesis and mitochondrial replacement techniques. Recent developments suggest that creating humans with predesignated phenotypes is imminent (70–73). The phrase ‘volitional evolution’ was introduced by Edward Osborne Wilson as ‘a species deciding what to do about its own heredity’ (70). To provide an ethical foundation, bioethicist S. Matthew Liao constructed a ‘human rights approach,’ which entitles all humans to certain fundamental conditions for pursuing a ‘good life’: those conducive to humans sustaining themselves corporeally, like food and water (71). Because inadequate space radiation protection results in deleterious health effects (e.g. cancer, cardiovascular and cognitive impairment, infertility, cataracts, etc.), the fundamental conditions for pursuing a good life are invalidated (74). Should it be possible to eliminate an offspring’s inherent radiosensitivity, Liao would assert that it can be impermissible not to do so (even defining non-life-threatening situations as worth consideration) (71, 75). However, jeopardizing an individual’s otherwise innate health based on risk alone poses an existential threat regarding the necessity of volitional evolution for this purpose. The ethics of allowing parents to irreversibly alter their child’s genome give rise to narrative identity and personal autonomy issues although are beyond the scope of this discussion.

## 3. Discussion: radioprotective transgenes

### 3.1 Strategies and complications

There are several genes known to confer radioprotection that may enhance survival after exposure to space radiation; the mechanisms of action for many are still not completely understood. The effects of introducing these genes into cells, animals and humans are function of both unknown and known variables, including inherent susceptibility and compatibility regarding genetic source and vector. For example, three forms of the enzyme superoxide dismutase (SOD) catalyze the conversion of superoxide into hydrogen peroxide (H_2_O_2_) in humans. SODs act as antioxidants by locally mitigating cellular reactive oxygen species (ROS), maintaining weight and survival probability when their genes are upregulated *in vivo* (76–78). Interestingly, differences in vector and route of administration manifest in outcome variations and heterogeneous expression levels of SOD specificity (79–86). Downstream of SOD, upregulating catalase enzyme further catalyzes the breakdown of H_2_O_2_ into water and oxygen, synergizing their radioprotection properties (87, 88). Separately, ascorbic acid has known antioxidant properties but is not naturally synthesized within humans; *Homo sapiens* harbor an evolutionarily conserved pseudogene instead of the encoding gene for L-gulonolactone oxidase, a precursor to ascorbic acid production (89, 90). The possibility of altering or bypassing the pseudogene to manufacture L-gulonolactone oxidase should be considered to augment the antioxidant capabilities of *Homo sapiens*.

**Figure 1.**
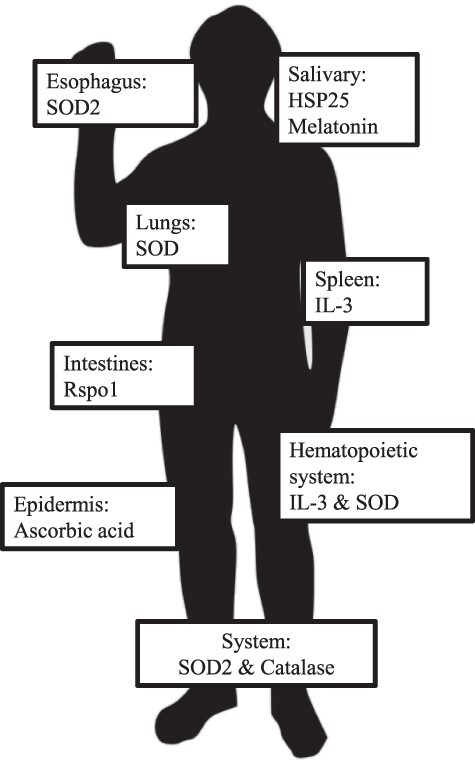
Local and systemic enzyme upregulation strategies for radioprotection and radiomitigation. These strategies should be combined with others for robust protection.

To enhance cellular antioxidant capacity, somatic strategies can confer purely localized benefits, such as those presented by heat shock protein 25 and melatonin on the salivary gland, or by SOD3 on the lungs (82, 91, 92). The local benefits of upregulating production of some enzymes are summarized in Figure 1. Interleukin-3 (IL-3), a cytokine showing transient benefit *in vivo* within the spleen and bone marrow, could be combined with other radioprotective agents for improved localization (86, 93). Roof plate-specific spondin-1 (Rspo1) has a proliferative effect on intestinal crypt cells in specific protecting the intestines and oral mucosa *in vivo* (94, 95). However, unlike the previous enzymes, Rspo1 acts as a radiomitigator instead of a radioprotector, reducing damage to normal tissues after radiation exposure, as opposed to prior to exposure (96). Given the known danger from space radiation, radioprotectors should be prioritized over their curative counterparts, with a combined therapy approach to provide the most comprehensive defense.

Although these proteins are naturally present in *Homo sapiens*, many so-called trans-species genes that confer radioprotection are found in other Animalia and other kingdoms of life. Organisms that overexpress DNA repair proteins exhibit augmented genome stability and enhanced mutagenic protection, with similar results when transplanted to mammalian cells (97–99). Fungal melanin has reduced cellular radiation effects when delivered *in vitro* and *in vivo* (100–105). The tardigrade’s damage suppressor protein (Dsup) halves double-stranded DNA breaks caused by photons in human cells (106–108). Despite native species potential, research model outcomes are clouded by interspecies differences in the target site sequence structure and DNA repair processes during gene therapy (109). If a gene proves ultimately incapable of interspecies transfer, expounding upon its mechanism of action may still elucidate novel directions for radioprotection (110).

### 3.2 Limitations in scope

Because symptoms of expedited aging result from radiation exposure, various geroprotectors have been proposed to suppress aging-related pathways, such as mTOR, ERK and p53 (111–113). Targeting components in the Ras/Raf/MEK/ERK pathway, for example, has validated clinical efficacy in slowing cancer progression and the effects of accumulated radiation exposure. Preclinical studies demonstrate MEK inhibition impacts proliferative, apoptotic and differentiation pathways downstream, potentially suppressing tumorigenicity (114, 115). However, convoluted downstream effects greatly complicate perceived clinical potential: increased Ras/Raf/MEK/ERK pathway expression decreases expression of the phosphatase and tensin homolog, contributing to carcinogenesis and prostate tumorigenesis (116, 117). Such corollary targets introduce incalculable collateral effects, the potential of which presently inhibits any clinical benefit. These conflicts are not exclusive to this pathway; the PI3K/PTEN/Akt/mTOR pathway has similar impediments to potential benefit in the field (118). Prior to tangible clinical progress from pathway alteration, a more comprehensive understanding of the downstream effects must be elucidated.

In addition to known limitations, unknown variables impede progress as well; the upregulation of these proteins has been investigated principally in the context of radiation therapy, conventionally analyzed within the milieu of isolated particle types of monoenergies (whereas other fields quintessentially lack radiation exposure) (119, 120). The default presence of background radiation levels over geological time scales during the evolutionary timeline introduces unanswered questions regarding acquired DNA repair mechanisms (121). Evidence suggests that epigenetic effects are relevant within this area, contributing to sustained beneficial traits at low dose-rate exposures. Controlled long-term experiments can shed light on the effects of constant background radiation in life’s evolution, possibly resulting in presently unknown amino acid sequences that confer safe and robust protection from GCR (122).

Should the practical (e.g. financial and technological) and ethical barriers to genetic engineering be superseded, the resultant radioprotection would likely not be comprehensive for the milieu of space, requiring shielding and other measures. Although difficult to speculate, even the most universal genetic solution could leave individuals vulnerable to spontaneous solar events, as well as the nebulous sequela of chronic GCR exposure. Indeed, the scarce information regarding chronic radiation exposure in humans is limited to biologically unpredictable heterogeneities (e.g. radiotherapy treatment) and immeasurable quantities (e.g. Chernobyl fallout) (123, 124). Still, epigenetic studies of large human cohorts with recognized exposure ranges (i.e. occupations or geographies with high background radiation) could aid in characterizing genotypes that confer radioresistance (125–127).

## 4. Discussion: alternatives to genetic alteration and modeling

### 4.1 Synergistic options

Considering such persistent limitations, supplemental or alternative approaches to genomic alterations need to be considered. Rocket propulsion, for example, is a constantly evolving field of study; it must inherently be improved to lessen journey time (and therefore decrease total radiation exposure). The commute and exposure time will be decreased when the chemical engine paradigm is replaced with that of electric propulsion (e.g. ion thrusters) (128). Modeling suggests that replacing the nuclear propulsion system with a purely electrical one would spare 1 year and 230 mSv on the roundtrip journey between Earth and Mars, requiring additional study (129). The present status of this endeavor and others are described in Table 2, alongside speculation regarding their feasibility. Another proposition confers radioprotection by enhancing pathways involved with sleep, as human cells have proven more susceptible to radiation damage after circadian interruption (130). Profound artificial depression of human metabolism into a synthetic torpor has been theorized to bypass these physiological challenges posed in deep space, although shallow states (defined as ∼20% below basal levels) have yet to be achieved in humans (131). Interestingly, suppressed metabolic activity is associated with condensed chromatin, which inherently confers heightened radioresistance to DNA (132, 133).

**Table 2.** Contemporary advantages, progress and predicted feasibility of various non-genetic strategies for augmenting the achievability of interplanetary human space endeavors

Strategy	Contemporary advantages	Existing research	Human employment feasibility (conjecture)
Optimize propulsion	Already well-established field of engineering (153)	Pragmatically, continuously (154)	Approach, but never achieve light speed, likely with nuclear thrusters
Synthetic torpor	Among most achievable in foreseeable future (155)	Philosophically (131)	Modest but perpetual metabolic depression on commute
Directed panspermia	May already take place on interplanetary scale, incidentally (134)	Philosophically (135)	Highly improbable
Synthetizing genome *sui genesis*	Rapidly growing field (138)	Mechanisms being explored (139)	Likely corollary to directed panspermia, limiting feasibility
Martian terraforming	Possibly achievable with existing technology (156)	Philosophically (157)	Highly improbable and opposed by NASA
Radiation-absorbing fungi	Already in existence (103)	Mechanisms being explored (105)	Cultivation as shielding probable, while genetic integration unlikely

Presently, the time necessary to traverse cosmic distances impedes the feasibility of corporeal human travel, requiring a broad range of possible solutions. Panspermia has been proposed as the possible origin of life on earth itself, and the essential environmental conditions needed for extraterrestrial habitation have been defined (134–137). This implicates the option to direct panspermia for human cell transmission to distant, hospitable planets. Meanwhile, the prospect of generating a synthetic human genome with chemicals to artificially manufacture human chromosomal DNA, and a whole-genome assembly may eventually be achieved by microarray-derived DNA oligonucleotides (which can already synthesize individual genes with limitations) (138–140). Although distributing synthetically constructed genes to probabilistically habitable planets is well-beyond current capabilities, it may be the most feasible option to avoid flight duration and radiation-based issues altogether. Terraforming the interior composition of the Martian planet itself could induce an artificial magnetosphere by the theorized dynamo mechanism or, alternatively, the atmosphere could conceivably be terraformed for physical shielding; however, terraforming of this magnitude is not condoned by NASA (141, 142).

### 4.2 Modeling and limitations

Although probabilistic scenarios may be estimated for an individual’s radiation exposure on a mission (for example, with Monte Carlo methodology), true physiological consequences remain ultimately unknown, especially combined with other effects from phenomena like microgravity and isolated environments (143). Without a comprehensive understanding of the phenotypic response of Homo *sapiens* to space radiation, a genotypic solution may misidentify or omit essential or corollary transcription pathways. Unforeseen issues may also manifest in execution, like abrupt and unsustainable germline or epigenetic mutations due to the unstudied synergistic effects of homology directed repair and chronic GCR exposure. NASA is attempting to isolate such issues by simulating simplified galactic cosmic rays at the NASA Space Radiation Laboratory at Brookhaven National Laboratory.

Machine learning has recently emerged as an applicable interdisciplinary tool to handle the dynamic nature of genes themselves, now modeled as statistical ensembles (144–146). Tasked with assembling spatial geometry from merely a sequence of amino acids, DeepMind’s AlphaFold (presently proprietarily owned by Google) achieved a watershed moment in 2020 for protein structure prediction (147, 148). AlphaFold remains the best predictor of tertiary structures, opening the possibility of reverse-engineering an optimized chain of amino acids provided a macroscopic structure (149, 150). Should a validated model emerge, *post hoc* machine learning could identify and evaluate likely downstream effects of targeted mutations (e.g. recognizing accidental off-site CRISPR effects). Although presently inconceivable due to intricate biochemical relationships, algorithms may eventually learn to synthesize amino acids into proteins *sui generis* to fit engineered applications, culminating in computer-generated cohorts (151, 152). Even if these proteins are not biologically feasible, integrating artificial intelligence with genetic engineering may facilitate computational modeling of otherwise overly complex biological networks, providing insight regarding cellular response to DNA modification.

### 4.3 Ethical considerations

While genetic modifications to decrease radiosensitivity to space radiation are transcendently intricate, existing technologies like preimplantation genetic diagnosis can already viably select children with preferred traits. S. Matthew Liao has suggested using this technology for a kindred quandary: to reduce the size of the population to mitigate anthropogenic climate change (158). The importance of considering such ostensibly radical ideas should not be ignored, as they serve as an important learning tool in stimulating revolutionary possibilities. Although the ideas vary tremendously in nature and severity, genomic engineering solutions are quintessentially constructed with technology harboring minimal risk and ample empirical study. Indeed, Liao argues that such risks should be weighed against those associated with taking inadequate action and notes that parents have the societal and biological right to reformulate their children, should doing so enhance well-being without alternative (159). We propose human gene engineering be considered and explored further in this debate regarding radiotolerance, while perpetuating transparency regarding potential dangers and merits.

## 5. Conclusions

Space radiation poses a formidable obstacle to humans in venturing beyond the protection of earth’s magnetosphere. Despite immense progress in the development and comprehension of CRISPR/Cas9-mediated gene editing in various model organisms, the efficiency and specificity with human cells must still be examined to a much greater depth (52). It is ethically unacceptable to inflict unpredictable and irreversible genomic effects upon humans without broad societal examination (56). However, should future generations embark upon prolonged extraterrestrial journeys, it may be unacceptable to forgo genetic tactics that may preserve their capability to enjoy a ‘good life.’ Considering the pervasiveness of space radiation and its physiological impacts, volitional evolution may confer the most robust solution, although parallel strategies should be deployed to provide comprehensive protection. We have described a number of supplemental strategies feasible for further consideration and have established an ethical foundation for their necessity within the context of danger from space radiation. We believe volitional evolution should be considered alongside other viable potential sources of radiation protection beyond low-Earth orbit.
